# Metal exposure from additive manufacturing and its effect on the nasal lavage fluid proteome - a pilot study

**DOI:** 10.1371/journal.pone.0256746

**Published:** 2021-08-31

**Authors:** Maria Assenhöj, Liam J. Ward, Bijar Ghafouri, Pål Graff, Stefan A. Ljunggren

**Affiliations:** 1 Department of Health, Medicine and Caring Sciences, Occupational and Environmental Medicine Center, Linköping University, Linköping, Sweden; 2 Department of Clinical Sciences, Intervention and Technology, Karolinska Institutet, Stockholm, Sweden; 3 Department of Health, Medicine and Caring Sciences, Pain and Rehabilitation Center, Linköping University, Linköping, Sweden; 4 National Institute of Occupational Health, Oslo, Norway; Government College University Faisalabad, Pakistan, PAKISTAN

## Abstract

The use of metal additive manufacturing (AM) is steadily increasing and is an emerging concern regarding occupational exposure. In this study, non-invasive sampled nasal lavage fluid (NLF) from the upper airways was collected from metal AM operators at the beginning and end of a workweek during two consecutive years with preventive interventions in the occupational setting in-between (n = 5 year 1, n = 9 year 2). During year one, NLF was also collected from welders (n = 6) from the same company to get a comparison with a traditional manufacturing technique with known exposure and health risks. The samples were investigated using untargeted proteomics, as well as using multi-immunoassay to analyze a panel of 71 inflammatory protein markers. NLF in AM operators from year 1 showed decreased levels of Immunoglobulin J and WAP four-disulfide core domain protein 2 and increased levels of Golgi membrane protein 1, Uteroglobin and Protein S100-A6 at the end of the workweek. At year two, after preventive interventions, there were no significant differences at the end of the workweek. In welders, Annexin A1 and Protein S100-A6 were increased at the end of the workweek. The analysis of 71 inflammatory biomarkers showed no significant differences between the beginning and the end of workweek year 1 in AM operators. We identified several proteins of interest in the AM operators that could serve as possible markers for exposure in future studies with a larger cohort for validation.

## Introduction

Additive manufacturing (AM) is a name given to techniques that produce components from the bottom up, often by creating them layer by layer [1]. The use of AM is steadily increasing due to their potential in producing advanced components not available with standard techniques, less material waste, and the possibility to create products in hard-to-reach places [2]. As such, there is an urgent need to fully understand the AM processes and the possible risks to operators being occupationally exposed [3].

Metal-based AM techniques commonly use alloys comprising of metals such as nickel, cobalt, chromium, aluminum, and titanium along with various stainless steels [4]. Since large-scale application of metal AM techniques provides a relatively new exposure scenario, there is little literature regarding exposure and health risk. However, several of the constituting metals are known to pose a health risk in other occupational settings. This includes that cobalt exposure decrease the lung function in hard metal workers [5] while nickel can cause lung diseases including asthma and damage the nasal cavity [6]. Nickel is also classified as a carcinogen Group 1 by IARC and has been linked to both cancer in the lungs, nasal cavity, and paranasal sinuses [7]. Furthermore, exposure-related lung function decline has been observed in steel workers exposed to a mix of cobalt, nickel, and chromium dust [8]. Welders constitute a large group of operators exposed to a mix of metals and have increased risk for lung diseases [9,10] and cardiovascular disease [11]. Some of the metals generated in AM are in the nanoscale range and therefore may constitute special risks to human health, similar to what has been shown for welders [12].

The upper airways constitute a major pathway of exposure for metals. It has been shown that inflammation and disease in the upper airways may predispose more serious lung complications [13] and that it may be a link between particles and cardiovascular disease [11]. Nasal lavage fluid (NLF) is collected by flushing the nasal cavity with a saline solution that allows the study of factors on the nasal mucous membrane. Due to its non-invasive nature, it is interesting for the use of finding markers for diseases and exposure including early markers of exposure that could precede eventual health effects. As such it has been applied to find changes in protein content (proteomic alterations) due to smoking [14] and allergic rhinitis [15] as well as in a range of occupational exposures including epoxy chemicals [16], trichloramine in swimming pool facilities [17], metalworking fluids [18], welding fumes [19], hairdressing chemicals [20] and chemical and biological agents released in moldy and damp buildings [21].

We have earlier described airborne and dermal metal exposure, as well as operators’ urinary metal concentrations, in one of the World´s first AM facility doing serial production using mainly nickel-cobalt alloy materials [22]. In the previous study, we found that the AM operators had a trend of higher metal concentrations in their urine and metals on their hands that were reduced after preventive interventions by the company, including powder-handling routines, ventilation, and personal protection equipment (PPE). The PPE that was introduced included a powered air purifying respirator with P3 particle filters and nitrile gloves taped to a coverall with protection against solid particles (per standard EN 13982–1) for all processes involving open powder handling. The same individuals have also been tested regarding different clinical tests and markers and an improved lung function and reduction of nickel in the blood after introduction of the preventive measures were identified [23]. In the present study, we analyzed the proteome of NLF from a small number of AM operators and welders described in the earlier studies [22,23] on Monday and Friday of a workweek to investigate effects of exposure in this novel and rapidly growing industry. Furthermore, we also sampled AM operators the subsequent year, after preventive interventions were implemented in their occupational setting, which would allow us to detect whether eventual proteomic alterations found during the first year were affected.

## Materials and methods

### Participants

**Fig 1:**
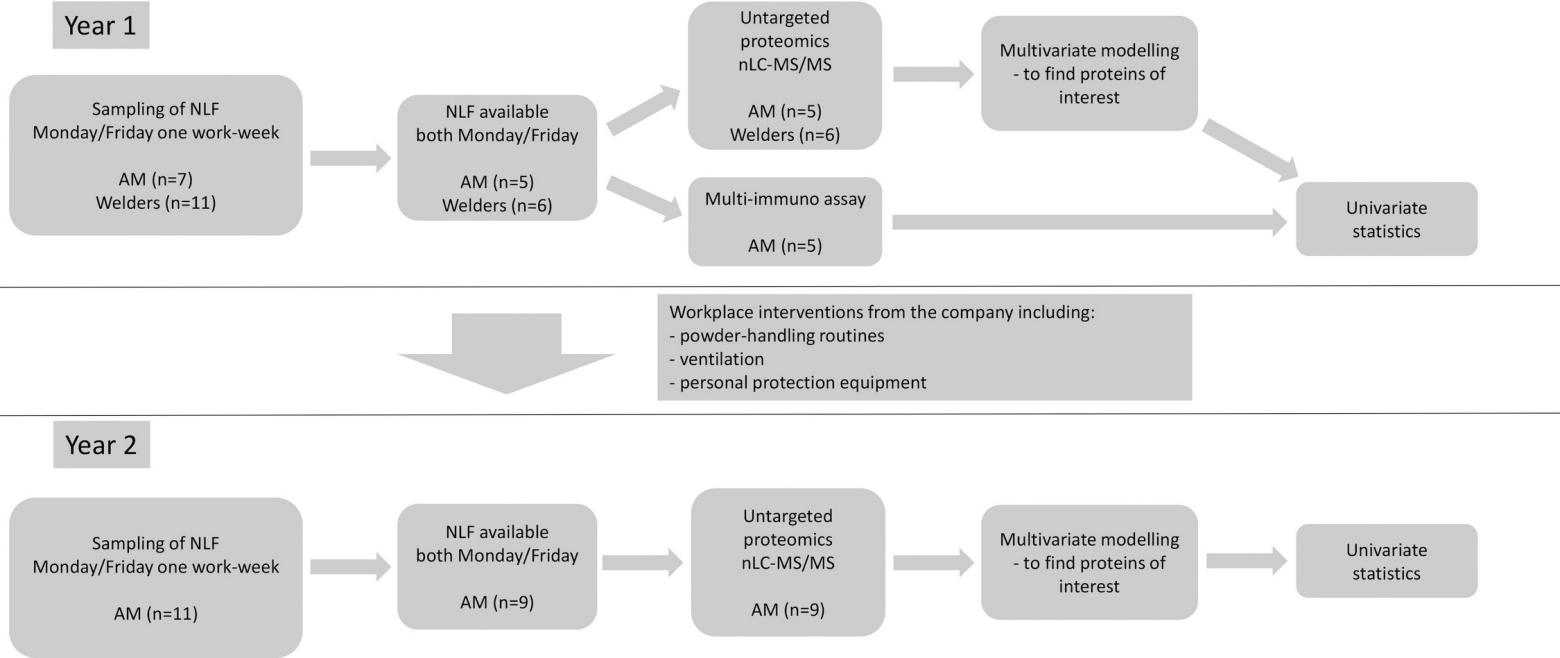
Flow-chart of study design.

Participants in the present study were the same as those earlier described [22]. In short, operators working at one of the world’s first facilities with serial manufacturing of metal AM components were recruited during two consecutive years, with preventive interventions to reduce the exposure in-between. During the first year, welders from the same company were also recruited to compare whether this traditional manufacturing technique with known exposure and health risks had similarities to operators working with AM. After signing an informed consent, participants provided NLF at the beginning and the end of a single workweek. Only individuals that had their NLF sampled both in the beginning and end of the week, each respective year, were included. Participants answered a survey regarding symptoms and whether they had any allergy or asthma. Participants’ height and weight were measured using a measuring tape mounted on a wall and a mechanical weight scale, respectively, to obtain their BMI. A flow-chart of the study design can be seen in Fig 1.

The study was approved by the Regional Ethics Board in Linköping with approval number 2016/112-31. A signed written informed consent was collected from all participants, which are stored at the Occupational and Environmental Medicine in Linköping.

### Nasal lavage fluid sampling

NLF was sampled during Monday and Friday for each participant. For logistic reasons, the time of sampling for each individual was spread over the course of the whole working day. To reduce any eventual effect of the circadian rhythm, each individual was sampled at similar time points on both days.

NLF was collected using a slightly modified protocol from previously published studies [18,21]. A catheter was placed in the subjects’ nostril and 10 mL of 0.9% NaCl were administered into the nasal cavity using a syringe and kept for 5 minutes before being collected. Samples were directly filtered through a mesh to remove mucus and subsequently centrifuged for 5 min at 4000G to remove cells. The supernatant was aliquoted and kept at -30°C.

### Nasal lavage fluid proteomics

Proteomics were performed similar to previously published research [24]. An aliquot of NLF was concentrated using 3 kDa spin filters (Merck Millipore, Burlington, MA, USA) and diluted with 8 M urea in 25 mM ammonium bicarbonate solution. Proteins were reduced and alkylated consecutively by incubating with 10 μL of 0.25M dithiothreitol and 0.75M iodoacetamide for 15 min at room temperature, respectively. The buffer was exchanged with 25 mM ammonium bicarbonate using the 3 kDa spin filter. Protein concentration was determined using 2-D Quant Kit (GE Healthcare, Little Chalfont, UK) and 10 μg of proteins were trypsinated (1:25, w/w trypsin/protein, Promega, Madison, WI, USA) overnight at 37°C. The peptides were dried using a SpeedVac vacuum concentration system (Savant, Farmingdale, NY, USA), reconstituted in 0.1% formic acid, and 0.25 μg was subjugated to nano liquid chromatography tandem mass spectrometry (nLC-MS/MS) analysis.

Peptides were separated by reverse-phase chromatography on an EASY-nLC II (Thermo Scientific, Waltham, MA, USA) by a linear gradient of 0.1% formic acid in water (A) and 0.1% formic acid in acetonitrile (B) (0–40% over 90 min). Automated online analyses were performed with an LTQ Orbitrap Velos Pro hybrid mass spectrometer (Thermo Scientific) with a nano-electrospray source.

Raw files were searched using MaxQuant v. 1.5.8.3 (Max Planck Institute of Bio-chemistry, Martinsried, Germany) against a UniProt Human database (downloaded 31st August 2018) with the following parameters: trypsin was used as digestion enzyme; maximum number of missed cleavages 2; fragment ion mass tolerance 0.50 Da; parent ion mass tolerance 6.0 ppm; fixed modification—carbamidomethylation of cysteine; variable modifications—N-terminal acetylation and a minimum of 2 peptides whereof at least 1 was unique. Data were filtered at a 1% false discovery rate. Proteins identified in 50% of the samples of individual groups were included in further analysis. Label-free quantification (LFQ) of protein level based on the total peptide intensity was done using the built-in LFQ algorithm.

### Multi-immunoassay analyses

The levels of 71 different inflammatory protein markers (cytokines/chemokines/growth factors) were measured using multi-immunoassay with the U-plex human 71-plex kit (Meso Scale Diagnostics, Rockville, MA, USA) in the NLF of AM operators Monday and Friday year 1. In brief, NLF was diluted according to the manufacturer´s instructions for the separate kits. Samples were added to the plates and incubated for 2 hours. After wash, detection antibodies were added and further incubated for 2 hours. After another wash, a read buffer was added, and markers were quantitatively measured via electrochemiluminescence using a MESO QuickPlex SQ 120. The lower levels of detection (LLOD) were determined using the Discovery Workbench 4.0 software using the mean of the background plus 2.5 times the standard deviation across all plates. Samples below the LLOD were imputed with LLOD/√2 for analysis.

### Statistical methods

Multivariate data analysis (MVDA) was performed on log10-transformed unit variance (UV) scaled, LFQ-intensity data in SIMCA (Sartorius Stedim Data Analytics AB, Umeå, Sweden) for the following groups individually; AM operators year 1, AM operators year 2 and Welders year 1. Variation in the data matrix was visualized using principal component analysis (PCA). The presence of strong and moderate multivariate outliers was investigated using Hotelling´s T2 and distance to model in X-space, respectively. No outliers were found. An orthogonal partial least squares-discriminant analysis (OPLS-DA) was used to investigate the correlation between NLF protein levels and group membership (Monday or Friday). Variables important for the separation of groups were selected using a variable influence on projection (VIP) value ≥ 1.2 and VIP value > standard error (SE). These proteins were subsequently included in a second OPLS-DA model with one predictive and one orthogonal component. Model quality was evaluated using R2 and Q2 describing the goodness of fit and prediction, respectively, and a CV-ANOVA.

Differences between groups regarding sex distribution, asthma or allergy diagnosis, age and were investigated by chi-square test and Mann-Whitney U-test, respectively. The non-parametric Wilcoxon matched-pairs signed-rank test was performed on nLC-MS/MS LFQ intensity data using Statistica (v13.5.0.17, TIBCO Software Inc) on proteins from the OPLS-DA MVDA models and multi-immunoassay proteins. A p-value < 0.05 was considered statistically significant.

## Results

### Participants

The number of individuals that participated both Monday and Friday were five AM operators year 1, nine AM operators year 2 and six welders year 1. There were no statistical differences between the groups regarding sex distribution, self-reported asthma/allergy, age, or BMI (Table 1). Daily symptoms from eyes or upper airways were also reported with cases of irritated, stuffy, or runny nose in AM operators year 2 and Welders year 1 (n = 2 and n = 3, respectively), blood-mixed nasal mucus/nosebleeds in AM operators year 1 and year 2 (n = 1 and n = 2, respectively), and irritated, itchy or stingy eyes in AM operators year 2 (n = 3).

**Table 1:** Participant characteristics.

	AM operators year 1 (n = 5)	AM operators year 2 (n = 9)	Welders year 1 (n = 6)
Sex (Males/Females)	4/1	6/3	6/0
Age (median, min-max)	42 (28–55)	37 (23–48)	43 (20–50)
BMI (median, min-max)	30.6 (22.7–31.2)	28.2 (22.7–31.9)	28.1 (24.9–29.5)
Smokers (yes/no)	0/5	2/7	2/4
Self-reported asthma diagnosis (yes/no)	1/4	2/7	3/3
Self-reported allergy diagnosis (yes/no)	3/2	3/6	2/4
Years worked in current occupational setting (no. of participants)			
<2	6	7	3
2–5	0	2	1
>5	0	0	2

AM: Additive manufacturing.

### Changes in protein levels during the workweek

Protein levels in Monday and Friday NLF-samples from year 1 in AM operators and welders, as well as year 2 in AM operators were analyzed. In total 171, 182 and 209 proteins were found in AM year 1, welders year 1 and AM year 2, respectively.

#### AM operators year 1

**Fig 2:**
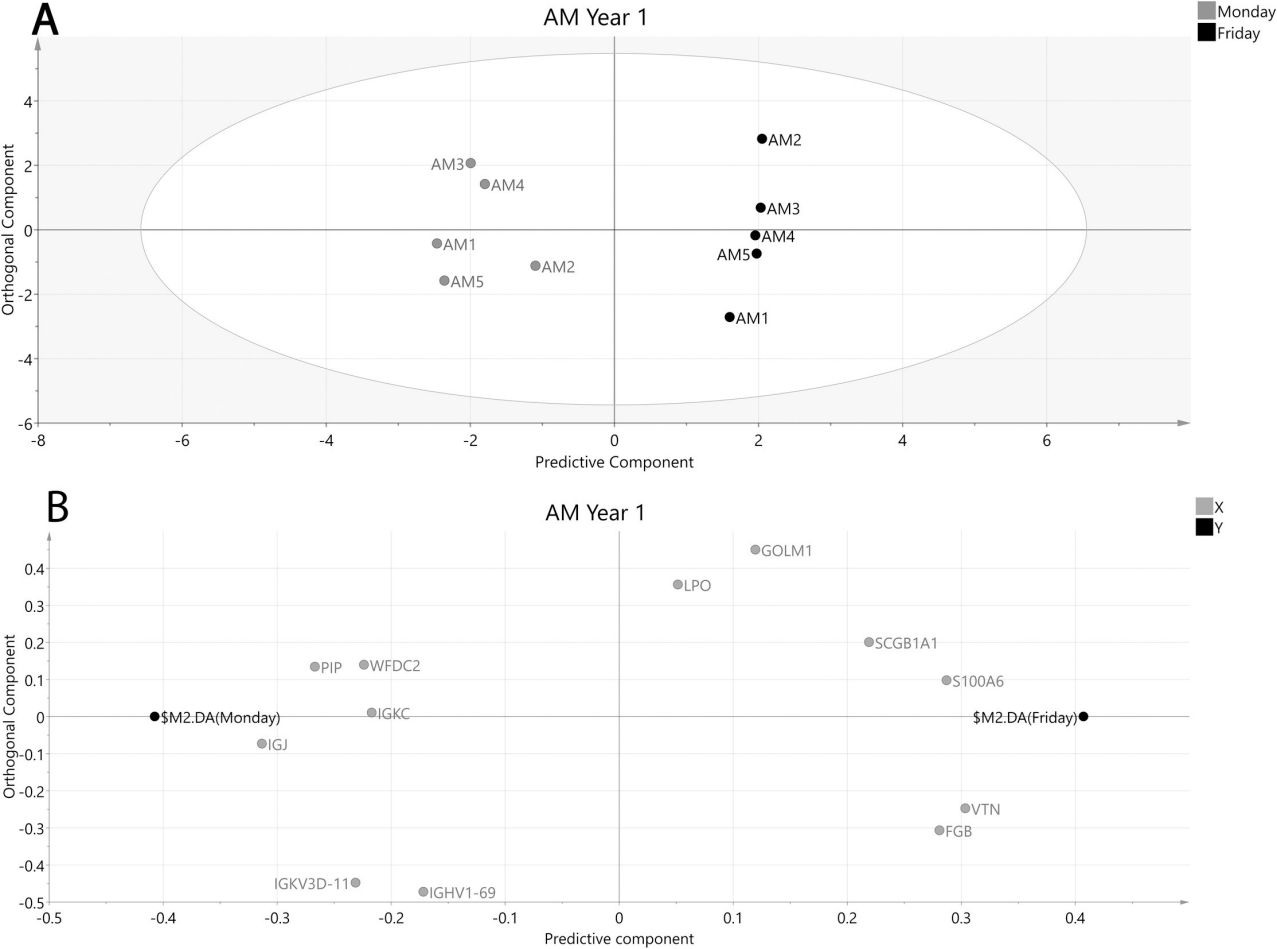
OPLS-DA model of AM operators year 1 after selecting proteins with VIP values ≥ 1.2 and VIP values > standard error (SE). A: Score plot of participating AM operators at the beginning and end of the week. The x-axis depicts the predictive component most important for separating samples collected Monday (grey circles) vs Friday (black circles) while the y-axis represent the orthogonal component showing in-group variation. R2 = 0.97, Q2 = 0.914, CV-ANOVA p-value = 0.007. B: Loading plot showing the underlying protein separation (X-variables, grey circles) in relation to sampling time point (Y-variables, black circles). FGB—Fibrinogen beta chain, GOLM1—Golgi membrane protein 1, IGHV1-69—Immunoglobulin heavy variable 1–69, JCHAIN—Immunoglobulin J chain, IGKC–Immunoglobulin kappa constant, IGKV3D-11 –Immunoglobulin kappa variable 3–11, LPO–Lactoperoxidase, PIP—Prolactin-inducible protein, S100A6—Protein S100-A6, SCB1A1 –Uteroglobin, WFDC2—WAP four-disulfide core domain protein 2, VTN–Vitronectin.

A MVDA was performed to see which differentially expressed proteins best described differences over a workweek on a group level. The generated MVDA model for AM operators year 1 (R2 = 0.97, Q2 = 0.91, CV-ANOVA p-value = 0.007) included 12 proteins and showed a clear separation between Monday and Friday samples (Fig 2).

Of the 12 proteins from the MVDA model, five were significant when investigated with univariate statistics (Table 2). Of these, Immunoglobulin J chain (Jchain) and WAP four-disulfide core domain protein 2 (Wfdc2) were decreased while Golgi membrane protein 1 (Golm1), Uteroglobin and Protein S100-A6 (S100-A6) were increased on Friday compared to Monday (Table 2).

**Table 2:** Proteins obtained from orthogonal partial least squares-discriminant analysis when comparing additive manufacturing operators Monday vs Friday year1.

Protein Name	Gene	UniProt Accession	Monday LFQ Intensity	Friday LFQ Intensity	Fold change Friday/Monday #	Protein Function (from UniProt)
Fibrinogen beta chain	*FGB*	P02675	1.3E+7 (1.2E+7–3.5E+7)	3.4E+7 (1.9E+7–4.4E+7)	2.6 (+)	Adaptive immunity, Blood coagulation, Innate immunity
Golgi membrane protein 1	*GOLM1*	Q8NBJ4	3.0E+6 (2.2E+6–5.8E+6)	4.9E+6 (2.5E+6–6.2E+6) *	1.6 (+)	Cellular protein metabolic process, Regulation of lipid metabolic process
Immunoglobulin heavy variable 1–69	*IGHV1-69*	P01742	2.1E+6 (1.5E+6–3.7E+6)	1.6E+6 (9.6E+5–2.9E+6)	0.8 (-)	B cell receptor signaling pathway, Complement activation, Innate immune response
Immunoglobulin J chain	*JCHAIN*	P01591	1.7E+8 (1.2E+8–2.8E+8)	9.7E+7 (6.6E+7–1.1E+8) *	0.6 (-)	Adaptive and innate immune response
Immunoglobulin kappa constant	*IGKC*	P01834	4.6E+7 (3.5E+7–6.1E+7)	3.3E+7 (1.5E+7–4.1E+7)	0.7 (-)	B cell receptor signaling pathway, Complement activation, Innate immune response
Immunoglobulin kappa variable 3–11	*IGKV3D-11*	P04433	2.5E+6 (1.4E+6–3.7E+6)	1.4E+6 (8.6E+5–2.7E+6)	0.6 (-)	Complement activation, Immune response
Lactoperoxidase	*LPO*	P22079	5.5E+6 (4.5E+6–9.8E+6)	5.9E+6 (5.6E+6–1.1E+7)	1.1 (+)	Hydrogen peroxide catabolic process, Response to oxidative stress
Prolactin-inducible protein	*PIP*	P12273	4.4E+8 (1.6E+8–7.1E+8)	2.2E+8 (1.6E+8–4.4E+8)	0.5 (-)	Negative regulation of T cell apoptotic process, Regulation of immune system process
Protein S100-A6	*S100A6*	P06703	1.7E+6 (1.2E+6–2.1E+6)	3.6E+6 (1.8E+6–6.4E+6) *	2.1 (+)	Positive regulation of fibroblast proliferation, Signal transduction
Uteroglobin	*SCGB1A1*	P11684	1.3E+7 (8.7E+6–3.1E+7)	2.3E+7 (1.9E+7–4.7E+7) *	1.8 (+)	Regulation of inflammatory response, signal transduction
WAP four-disulfide core domain protein 2	*WFDC2*	Q14508	4.7E+7 (3.5E+7–8.5E+7)	2.9E+7 (2.4E+7–5.6E+7) *	0.6 (-)	Proteolysis
Vitronectin	*VTN*	P04004	1.9E+5 (7.3E+4–3.6E+5)	4.9E+5 (3.0E+5–6.0E+5)	2.6 (+)	Immune response. Cell adhesion, Regulation of Complement activation

Values are median (range), LFQ–label free quantification. # Sign in parenthesis depicts increase (+) or decrease (-) over the workweek.

* p<0.05 Wilcoxon matched pairs test when comparing Monday vs Friday samples.

#### AM operators year 2

**Fig 3:**
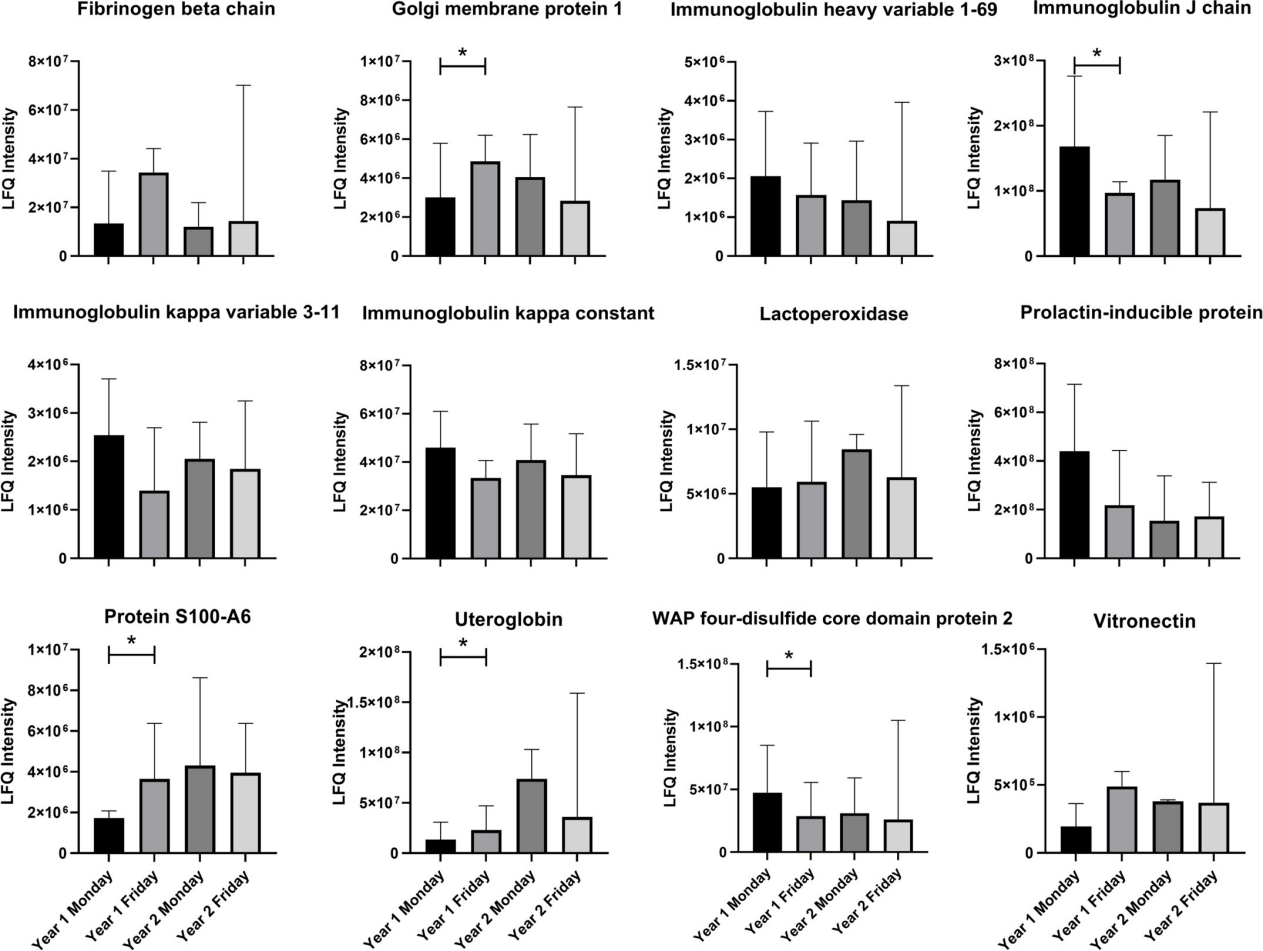
Comparison of protein differences in nasal lavage fluid collected from additive manufacturing operators year 1 and year 2. Values are median (range), LFQ–label free quantification, * p<0.05 Wilcoxon matched pairs test.

The generated MVDA model for AM operators could not separate Monday and Friday samples for year 2, and all the quality parameters were too low to be able to use the model for further analysis (R2 = 0.61, Q2 = 0.25, p > 0.05, S1 Fig). When comparing the proteins obtained in the MVDA model from year 1 (Table 2) with the samples from year 2, none showed any statistical significance (Fig 3).

#### Welders year 1

**Fig 4:**
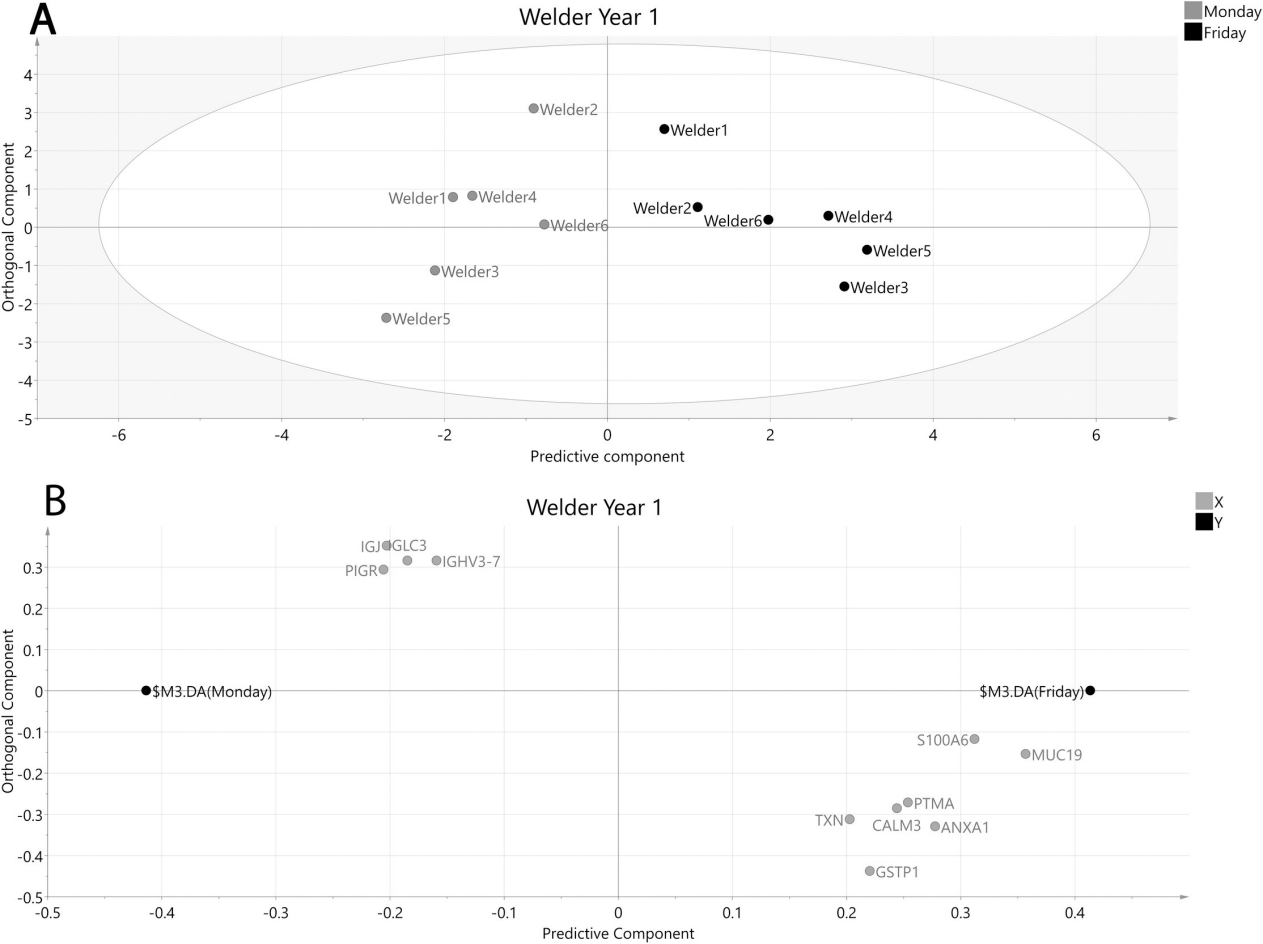
OPLS-DA model of welders year 1 after selecting proteins with VIP values ≥ 1.2 and VIP values > standard error (SE). A: Score plot of participating welders at the beginning and end of the week. The x-axis depicts the predictive component most important for separating samples collected Monday (grey circles) vs Friday (black circles) while the y-axis represent the orthogonal component showing in-group variation. R2 = 0.84, Q2 = 0.20, p > 0.05. B: Loading plot showing the underlying protein separation (X-variables, grey circles) in relation to sampling time point (Y-variables, black circles). ANXA1—Annexin A1, CALM3—Calmodulin-3, GSTP1—Glutathione S-transferase P, IGHV3-7—Immunoglobulin heavy variable 3–7, IGJ—Immunoglobulin J chain, IGLC3—Immunoglobulin lambda constant 3, MUC19—Mucin-19, PIGR—Polymeric immunoglobulin receptor, PTMA—Prothymosin alpha, S100A6—Protein S100-A6, TXN–Thioredoxin.

In welders, MVDA modeling resulted in 11 proteins separating Monday and Friday samples although the model had poor predictive quality and was non-significant for CV-ANOVA (R2 = 0.84, Q2 = 0.20, p > 0.05, Fig 4).

Of the 11 proteins being modestly separated in welders Monday vs Friday year 1, two were found significant by univariate statistics; Annexin A1 (AnxA1) and Protein S100-A6 (S100-A6) were increased on Friday compared to Monday (Table 3).

**Table 3:** Proteins obtained from orthogonal partial least squares-discriminant analysis when comparing welders Monday vs Friday year1.

Protein Name	Gene	UniProt Accession	Monday LFQ Intensity	Friday LFQ Intensity	Fold change Friday/Monday #	Protein Function (from UniProt)
Annexin A1	*ANXA1*	P04083	2.4E+6 (1.8E+6–2.6E+6)	3.1E+6 (2.1E+6–5.8E+6) *	1.3 (+)	Inflammatory response–innate and adaptive
Calmodulin-3	*CALM3*	P0DP25	5.3E+5 (2.7E+5–1.2E+6)	1.6E+6 (7.7E+5–3.2E+6)	3.0 (+)	Calcium-mediated signaling. G protein-coupled receptor signaling pathway
Glutathione S-transferase P	*GSTP1*	P09211	2.6E+6 (1.3E+6–2.8E+6)	3.4E+6 (1.9E+6–5.2E+6)	1.3 (+)	Glutathione metabolic process. negative regulation of acute inflammatory response
Immunoglobulin heavy variable 3–7	*IGHV3-7*	P01780	2.8E+6 (1.6E+6–8.7E+6)	1.7E+6 (1.2E+6–3.8E+6)	0.6 (-)	B cell receptor signaling pathway. Complement activation. Innate immune response
Immunoglobulin J chain	*JCHAIN*	P01591	1.2E+8 (9.6E+7–1.9E+8)	9.1E+7 (5.1E+7–1.2E+8)	0.8 (-)	Adaptive and innate immune response
Immunoglobulin lambda constant 3	*IGLC3*	P0DOY3	2.8E+8 (1.8E+8–3.8E+8)	2.1E+8 (1.8E+8–2.4E+8)	0.7 (-)	B cell receptor signaling pathway. Complement activation. Innate immune response
Mucin-19	*MUC19*	Q7Z5P9	2.4E+7 (2.1E+7–4.3E+7)	5.3E+7 (4.0E+7–1.0E+8)	2.2 (+)	O-glycan processing
Polymeric immunoglobulin receptor	*PIGR*	P01833	4.4E+8 (3.3E+8–5.2E+8)	3.2E+8 (2.3E+8–4.7E+8)	0.7 (-)	Fc receptor signaling pathway. neutrophil degranulation
Prothymosin alpha	*PTMA*	P06454	1.2E+5 (9.1E+4–1.9E+5)	5.5E+5 (2.0E+5–8.3E+5)	4.4 (+)	Negative regulation of apoptotic process
Protein S100-A6	*S100A6*	P06703	2.1E+6 (1.2E+6–3.0E+6)	4.7E+6 (1.8E+6–6.0E+6) *	2.2 (+)	Positive regulation of fibroblast proliferation; Signal transduction
Thioredoxin	*TXN*	P10599	6.6E+5 (2.4E+5–1.7E+6)	1.4E+6 (6.2E+5–4.5E+6)	2.1 (+)	Oxidation-reduction process

Values are median (range), LFQ–label free quantification. # Sign in parenthesis depicts increase (+) or decrease (-) over the workweek.

* p<0.05 Wilcoxon matched pairs test when comparing Monday vs Friday samples.

### Multi-immunoassay

The levels of 71 inflammatory proteins in NLF were investigated using multi-immunoassay. None of the investigated proteins showed any significant changes between Monday and Friday in the AM operators year 1 (S1 Table).

## Discussion

NLF constitutes an interesting matrix to identify early markers of exposure in the upper airways that could precede health effects. As such, it has previously been used in chamber studies of welding fumes [19] but is entirely novel for the much less studied AM environment. In this pilot study, the protein levels in NLF were investigated in AM operators and welders at two time points during a single workweek over two consecutive years. As such, the participating individuals functioned as their own controls during each workweek with regard to eventual exposure-related effects to alleviate eventual inter-individual differences, including possible non-exposure related factors that could affect the NLF including asthma or allergy. We identified protein changes during year 1 in the AM operators, but not year 2 after preventive interventions that reduced the occupational exposure in the environment had been implemented [22,23]. This indicates that the identified proteins in NLF were likely exposure-related and therefore of interest as markers of metal AM exposure.

### Proteins with decreased levels after a workweek

Immunoglobulin J (Jchain) functions as a linking molecule for monomers of IgA or IgM and therefore serves a role in adaptive immunity. Jchain was found decreased in AM operators over the workweek year 1. Previously a similar decrease has been shown in workers with airway symptoms exposed to metalworking fluids compared to individuals not showing airway symptoms [17]. Jchain has also been found to be decreased in the nasal mucosa of individuals with allergic rhinitis as compared to healthy controls [25]. This indicates that the reduced level identified in the AM operators may be of clinical significance as a marker of developing upper airway disease. In addition, levels of the Jchain gene increases in whole blood due to cigarette smoke [26], indicating that both gene and protein could be of major interest regarding exposure. Although the relationship between short and long-term exposure needs to be further studied.

WAP four-disulfide core domain protein 2 (Wfdc2) is an endopeptidase inhibitor known to be expressed in the respiratory tract. An earlier study of NLF from welders showed a decrease of Wfdc2 after controlled chamber exposure to welding fumes [19], similar to what we found in AM operators during year 1. Like Jchain, Wfdc2 was shown to be decreased in the nasal mucosa of individuals with allergic rhinitis as compared to healthy controls [25]. Therefore, Wfdc2 together with Jchain may have an interesting clinical significance in the nasal mucosa.

### Proteins with increased levels after a workweek

Golgi membrane protein 1 (Golm1) is a protein with yet unknown function that has been shown to increase in viral infections. Golm1 is increased in adenocarcinoma tissues as well as in serum of lung cancer patients [27]. It has also been indicated as a possible immune-modulating agent by selective inhibition of interleukin-12 production in dendritic cells [28]. Golm1 was found to be increased Friday in the AM operators year 1, indicating an effect on the immune response in the nasal cavity of the AM operators.

Protein S100-A6 (S100-A6) is a calcium sensor and modulator that is increased in cancer tissues and proposed as a prognostic marker for lung cancer [29,30]. Furthermore, S100-A6 has been shown to be increased in rat bronchial epithelial cells and lung tissues, after exposure to cigarette smoke. It was suggested that S100-A6 may act as a potential trigger, probably by binding to the receptor for advanced glycation end products (RAGE), for the intracellular signal cascade in the process of smoke-related inflammation [31]. In NLF, S100-A6 was increased Friday in both AM operators and welders in year 1, which may indicate a shared pathway driven by metal exposure that may be of significance for inflammation in the nasal cavity. It is therefore tempting to suggest that S100-A6 may be a marker for metal exposure, regardless of whether it is welding or AM, in the NLF. It should be noted that there is a difference in exposure between the AM and the welding environments where the former have predominantly larger particles (μm in size) compared to nanoparticles in the latter [19,22]. The effect of exposure to solely nanoparticles vs larger particles on the NLF proteome is however not known. Furthermore, it is also likely that the metal constituents of the particles are important to determine an eventual effect in the NLF. This could eventually be a reason why we did not identify similar protein changes in the welders as in a previously published chamber study with welding fume exposure [19], although other explanations including differences in particle doses, length of the exposure, and number of participants or the presence of other exposures in our actual welding environment may also be contributing. Altogether the prospect of S100-A6 as a general exposure marker as well as the influence of different particles sizes and compositions on the NLF proteome warrant further investigations.

Uteroglobin is a phosphatidylcholine/phosphatidylinositol binding protein that functions as a potent inhibitor of phospholipase A2. As such, Uteroglobin regulates interleukin production and thereby the inflammatory response. Uteroglobin is highly expressed in Club cells (also called Clara cells) in the airways and is also called Clara cell protein (CC10 or CC16). Uteroglobin is described as an anti-inflammatory protein and reduced serum levels of it has been described as both a marker and possible mechanism for the progression of airway disease [32,33]. Furthermore, Uteroglobin is reduced in the bronchial fluid after exposure to diesel exhaust, although with no effect on levels in NLF [34]. The underlying anti-inflammatory molecular mechanism may involve its ability to reduce the activation of NF-κB [35] as well as modulating dendritic cells, which inhibits TH17 and TH2 cells responses [36]. Uteroglobin has earlier been shown to be increased in welders exposed to controlled welding fumes in a chamber [19]. Similarly, we found that AM operators during year 1 had higher levels of Uteroglobin Friday vs Monday. This may reflect activation of a compensatory mechanism and an increased need for the anti-inflammatory properties of Uteroglobin, similar to what has been hypothesized in rats [37].

In welders, we found an increase of Annexin A1 (AnxA1), also called Lipocortin I. AnxA1 is increased in plasma of patients with chronic obstructive pulmonary disease, and in vitro cigarette smoke causes increased levels and an effect on lung fibroblast function [38]. AnxA1 is described as an anti-inflammatory protein and has previously shown to be increasingly cleaved in NLF due to upper airway disease and smoking [14,39]. Similar to Uteroglobin for AM operators, the increase of AnxA1 may depend on an increased need for anti-inflammatory effect in the nasal cavity.

### Protein markers in NLF

In contrast to the proteins detected using nLC-MS/MS, the 71 protein markers measured in NLF with multi-immunoassay showed no significant changes. These markers are part of a human biomarker group including cytokines, chemokines and interferons that are targeted at plasma, serum, or cell supernatants. It has been shown that proteins may exudate from plasma to the airway epithelial layer in the presence of inflammatory mediators such as in allergy [40]. The nasal cavity thereby represents a complex milieu where proteins may be either cell- or plasma-derived, of which the latter is, likely not a constant mechanism that may be activated based on tissue-dwelling cells in the epithelial lining [41]. Our results indicate that there either is no inflammation occurring in the upper airways of the investigated operators, or that the investigated protein markers may not be of interest in the NLF in this type of occupational setting. However, our untargeted proteomics analyses indicated proteins involved in immune responses and the 71 protein markers were chosen based on their presence in plasma and cells while the levels of the proteins in the NLF is not known. As such, there could still be inflammation occurring in the upper airways or even a systemic inflammation that needs to be further studied. Of note, several of the proteins markers appeared as if they were decreasing during the workweek. The reason for this is not known but should be addressed in a larger study.

### Limitations and future perspectives

This pilot study is limited to a small number of participating individuals. As earlier described [22], we recruited all available AM operators at the company for participation in the study. However, for NLF, we had one AM operator that declined participation in both years and one that only participated Monday year 1. The small size of the present study is a drawback but as described in the initial paper [22], the AM facility in which participants work is one of the world-leading sites using AM for actual serial production of metal components. Due to the fast increase in AM applications worldwide, it is of importance to investigate the effects of exposure, even if the number of participating individuals is relatively few. Thus, this study serves as a pilot study and starting point for future investigations to occupational risks with AM that need to be conducted to illuminate whether there are long-term health effects in these types of facilities. It would be beneficial in future studies to include a non-exposed population for comparison of the NLF proteome at the end of the workweek. Another aspect worth pursuing is a thorough determination of how different particle sizes and composition affects the NLF proteome using well controlled exposures and then compare these with actual occupational exposures.

## Conclusions

In summary, the present pilot study shows that exposed AM operators have alterations in their NLF proteome related to workweek exposure in the facility. These proteins were mainly involved in immune responses and could signify an active immune reaction in the upper airways. These alterations were not found in year 2 after preventive interventions at the workplace, including powder-handling routines, ventilation, and personal protection equipment that reduced the exposure [22]. Until now, there have been no long-term studies of health effects in AM operators due to the relatively few individuals that have been working with AM on a larger scale. However, our study highlights the need to pro-actively reduce the exposure to remove effects in the nasal cavity and therefore hopefully minimize the risk for occupational airway disease in AM operators.

## Supporting information

S1 Fig OPLS-DA model of AM operators year 2. (DOCX)

S1 Table Level of 71 biomarkers in NLF of AM operators investigated with multi-immunoassay. (DOCX)

S1 File Data underlying tables and figures in the manuscript. (XLSX)
