# Supplementary material for: Metal exposure from additive manufacturing and its effect on the nasal lavage fluid proteome - a pilot study
Source: PLoS One. 2021 Aug 31;16(8):e0256746. doi: 10.1371/journal.pone.0256746 (PMC8407577; doi:10.1371/journal.pone.0256746)
Supplement: S1 Fig — (DOCX) [file pone.0256746.s001.docx]

# **Supplementary Figure 1**


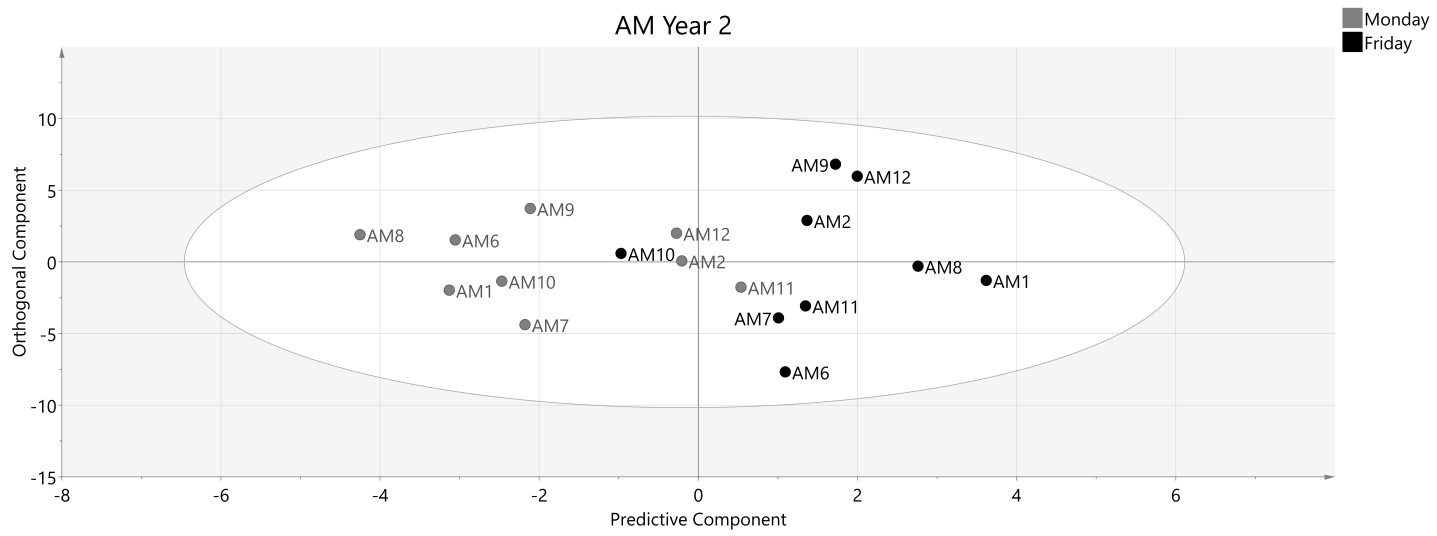


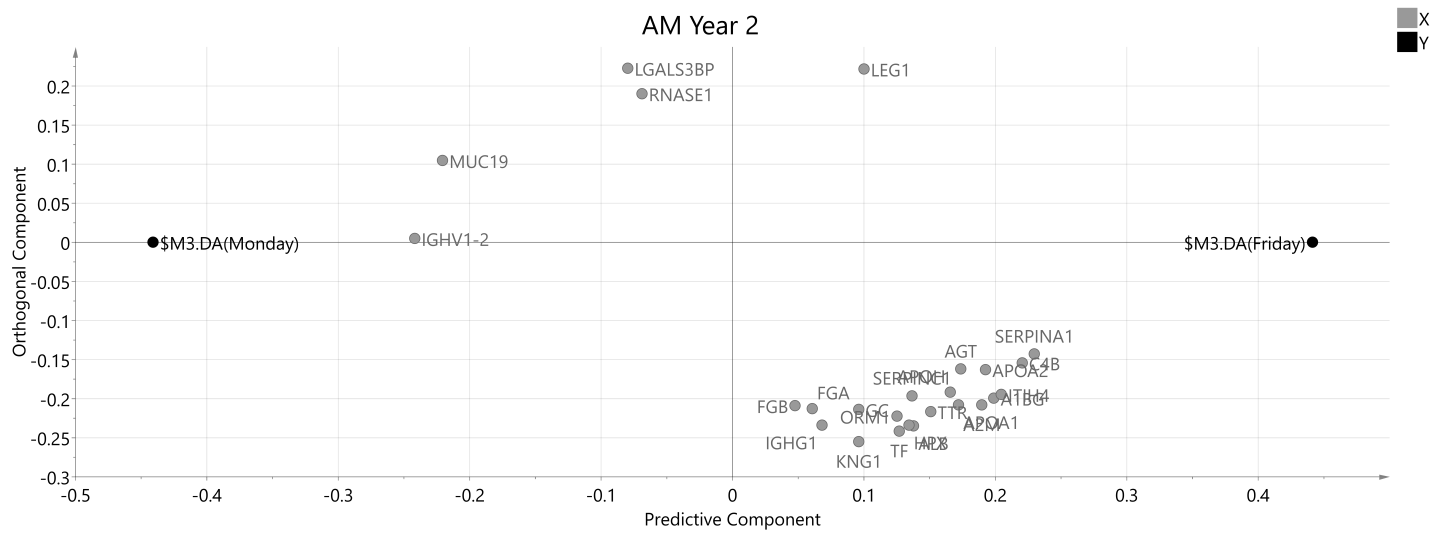


S1 Fig. OPLS-DA model of AM operators year 2 after selecting proteins with VIP values ≥ 1.2 and VIP values > standard error (SE). Upper panel: Score plot of participating welders at the beginning and end of the week. The x-axis depicts the predictive component most important for separating samples collected Monday (grey circles) vs Friday (black circles) in the welders while the y-axis represent the orthogonal component showing in-group variation. R2 = 0.61, Q2 = 0.25, p > 0.05. Lower panel: Loading plot showing the underlying protein separation (X-variables, grey circles) in relation to sampling time point (Y-variables, black circles).
