# Supplementary material for: Metal exposure from additive manufacturing and its effect on the nasal lavage fluid proteome - a pilot study
Source: PLoS One. 2021 Aug 31;16(8):e0256746. doi: 10.1371/journal.pone.0256746 (PMC8407577; doi:10.1371/journal.pone.0256746)
Supplement: S1 Table — (DOCX) [file pone.0256746.s002.docx]

**S2_table. Level of 71 biomarkers in NLF of AM operators investigated with multi-immunoassay**

| Biomarker (pg/mL) | Monday (n=5) | Friday (n=5) | Biomarker (pg/mL) | Monday (n=5) | Friday (n=5) |
| --- | --- | --- | --- | --- | --- |
| CTACK | 0.5 (0.5-5.6) | 0.5 (0.5-0.8) | **IL-27** | 10.2 (10.2-17.3) | 10.2 (10.2-10.2) |
| ENA-78 | 3.3 (0.2-71.4) | 1.9 (0.8-568.3) | **IL-29/IFN-L1** | 0.2 (0.2-0.7) | 0.2 (0.2-0.6) |
| Eotaxin | 8.1 (8.1-96.8) | 8.1 (8.1-38.8) | **IL-2Ra** | 14.8 (14.8-41.8) | 14.8 (14.8-14.8) |
| Eotaxin-2 | 26.8 (2.8-169.0) | 31.3 (8.1-142.0) | **IL-3** | 9.1 (9.1-18.4) | 9.1 (9.1-9.1) |
| Eotaxin-3 | 10.4 (2.7-50.1) | 5.3 (2.7-9.3) | **IL-31** | 13.6 (13.6-13.6) | 13.6 (13.6-13.6) |
| EPO | 1.3 (1.3-2.9) | 1.3 (1.3-1.3) | **IL-33** | 0.3 (0.3-0.3) | 0.3 (0.3-0.3) |
| FLT3L | 10.8 (0.4-13.6) | 10.5 (0.1-11.9) | **IL-4** | 1.0 (0.0-1.5) | 0.3 (0.0-1.9) |
| Fractalkine | 111.0 (20.3-721.9) | 112.4 (20.3-272.4) | **IL-5** | 0.9 (0.2-1.3) | 0.4 (0.1-3.3) |
| G-CSF | 1212.9 (15.5-13791.2) | 167.9 (4.9-17418.6) | **IL-6** | 211.9 (0.1-2328.1) | 49.4 (0.1-3094.9) |
| GM-CSF | 320.6 (0.0-4190.7) | 80.8 (0.0-3401.0) | **IL-7** | 2.0 (0.7-13.3) | 1.8 (0.3-9.6) |
| GRO-α | 4966.1 (31.0-5726.7) | 4536.8 (50.8-6194.4) | **IL-8** | 3130.3 (56.7-3227.5) | 1509.1 (5.3-3118.8) |
| I-309 | 12.9 (2.6-41.3) | 6.5 (2.6-44.4) | **IL-9** | 0.1 (0.1-0.8) | 0.1 (0.1-0.1) |
| IFN-α2a | 1.0 (0.8-2.4) | 0.5 (0.5-2.4) | **IP-10** | 20.7 (0.5-2537.6) | 19.4 (5.5-873.3) |
| IFN-ß | 1671.2 (590.8-2401.8) | 2109.9 (542.1-2349.2) | **I-TAC** | 1.6 (1.6-86.5) | 1.6 (1.6-72.9) |
| IFN-γ | 9.7 (1.8-27.8) | 15.2 (1.8-34.7) | **MCP-1** | 262.1 (1.0-849.7) | 209.8 (1.0-4975.3) |
| IL-10 | 1.4 (0.1-2.0) | 0.4 (0.0-11.6) | **MCP-2** | 0.4 (0.1-1.8) | 0.3 (0.1-1.6) |
| IL-12/IL-23p40 | 2.0 (0.7-2.8) | 1.5 (0.7-4.6) | **MCP-3** | 6.4 (0.7-12.7) | 5.8 (0.7-17.7) |
| IL-12p70 | 2.6 (0.2-4.4) | 1.7 (0.2-7.5) | **MCP-4** | 13.5 (13.5-134.1) | 13.5 (13.5-82.3) |
| IL-13 | 7.1 (2.3-18.1) | 4.8 (2.3-11.9) | **M-CSF** | 33.7 (0.5-45.9) | 39.7 (0.1-81.8) |
| IL-15 | 2.6 (0.2-3.7) | 3.1 (0.2-3.7) | **MDC** | 34.5 (34.5-151.1) | 34.5 (34.5-34.5) |
| IL-16 | 2.1 (1.4-22.8) | 1.4 (1.4-53.9) | **MIF** | 27109.3 (590.6-28151.1) | 14514.5 (985.1-28853.7) |
| IL-17A | 0.9 (0.9-11.3) | 0.9 (0.9-9.1) | **MIP-1a** | 11.0 (6.2-45.2) | 6.2 (6.2-26.4) |
| IL-17A/F | 1.3 (1.3-9.5) | 1.3 (1.3-5.1) | **MIP-1ß** | 2.2 (1.1-11.5) | 1.1 (1.1-7.1) |
| IL-17B | 0.7 (0.7-10.7) | 0.7 (0.7-0.7) | **MIP-3a** | 63.0 (1.9-1197.8) | 36.1 (1.9-1264.5) |
| IL-17C | 3.2 (0.6-3.8) | 0.6 (0.6-11.8) | **MIP-3ß** | 1.5 (1.5-108.0) | 1.5 (1.5-120.9) |
| IL-17D | 17.1 (17.1-17.1) | 17.1 (17.1-17.1) | **MIP-5** | 0.9 (0.6-815.0) | 1.2 (0.4-1240.7) |
| IL-17E/IL-25 | 0.4 (0.4-1.8) | 0.4 (0.4-0.4) | **SDF-1alpha** | 102.7 (102.7-533.7) | 102.7 (102.7-448.5) |
| IL-17F | 47.8 (47.8-222.6) | 47.8 (47.8-47.8) | **TARC** | 0.1 (0.1-13.1) | 0.1 (0.1-5.8) |
| IL-18 | 17.7 (6.2-23.1) | 9.1 (6.7-36.1) | **TNF-a** | 3.7 (0.3-33.0) | 0.5 (0.3-43.6) |
| IL-1RA | 798.5 (470.4-4975.5) | 753.3 (236.6-3494.9) | **TNF-ß** | 0.2 (0.2-0.6) | 0.2 (0.2-0.2) |
| IL-1a | 305.4 (1.1-415.3) | 82.8 (4.9-425.1) | **TPO** | 2.4 (2.4-4.8) | 2.4 (2.4-2.4) |
| IL-1ß | 13.2 (0.5-24.8) | 4.9 (0.4-19.7) | **TRAIL** | 2.4 (0.4-517.1) | 1.9 (1.0-749.8) |
| IL-2 | 5.5 (0.2-10.0) | 1.0 (0.2-9.7) | **TSLP** | 3.8 (1.7-89.0) | 1.8 (1.7-117.8) |
| IL-21 | 8.4 (8.4-8.4) | 8.4 (8.4-8.4) | **VEGF-A** | 1360.9 (35.2-2030.4) | 955.8 (2.1-1427.7) |
| IL-22 | 0.1 (0.1-0.2) | 0.1 (0.1-5.9) | **YKL-40** | 158.6 (20.6-1219.9) | 194.5 (8.1-5484.7) |
| IL-23 | 0.6 (0.6-2.4) | 0.6 (0.6-0.6) |  |  |  |

Values are median (range). CSF – colony-stimulating factor, INF – interferon, IL – Interleukin, MCP – monocyte chemoattractant protein, MIP – Macrophage inflammatory protein, TNF – tumor necrosis factor.
